# Serum steroid profiling of hepatocellular carcinoma associated with hyperadrenocorticism in dogs: A preliminary study

**DOI:** 10.3389/fvets.2022.1014792

**Published:** 2022-09-28

**Authors:** Thandar Oo, Noboru Sasaki, Yoshinori Ikenaka, Takahiro Ichise, Noriyuki Nagata, Nozomu Yokoyama, Kazuyoshi Sasaoka, Keitaro Morishita, Kensuke Nakamura, Mitsuyoshi Takiguchi

**Affiliations:** ^1^Laboratory of Veterinary Internal Medicine, Department of Veterinary Clinical Sciences, Graduate School of Veterinary Medicine, Hokkaido University, Sapporo, Japan; ^2^Veterinary Teaching Hospital, Faculty of Veterinary Medicine, Hokkaido University, Sapporo, Japan; ^3^Laboratory of Toxicology, Department of Environmental Veterinary Sciences, Faculty of Veterinary Medicine, Hokkaido University, Sapporo, Japan

**Keywords:** adrenocortical hormones, hepatocarcinogenesis, baseline serum, LC/MS/MS, dogs

## Abstract

**Background:**

Hepatocellular carcinoma (HCC) is one of the most common primary liver tumors in humans and dogs. Excessive adrenocortical hormone exposure may cause steroid hepatopathy, which may develop into HCC. In our previous study, hyperadrenocorticism (HAC) was a highly concurrent disease in dogs with HCC. Therefore, this study hypothesized that adrenal steroid alterations might be involved in hepatocarcinogenesis and aimed to specify the relationship between HAC and HCC in dogs.

**Materials and methods:**

This study included 46 dogs brought to the Hokkaido University Veterinary Teaching Hospital between March 2019 and December 2020. Owners gave their signed consent for blood collection on their first visit. A total of 19 steroids (14 steroids and 5 metabolites) in the baseline serum of 15 dogs with HCC, 15 dogs with HAC, and 10 dogs with both diseases were quantitatively measured using the developed liquid chromatography coupled with tandem mass spectrometry (LC/MS/MS) method.

**Results:**

In each group, 11 steroids were detected higher than 50%. The detection rate of steroid hormones did not significantly differ between the groups (*p* > 0.05). Principle component analysis (PCA) showed that the steroid profiles of the three groups were comparable. Median steroid hormone concentrations were not significantly different between the study diseases (*p* > 0.05).

**Conclusion:**

The developed LC/MS/MS was useful for measuring steroid hormones. Although it was clear that HAC was concurrent in dogs with HCC, none of the serum steroids was suggested to be involved in HCC.

## Introduction

The hepatocellular tumor is a common hepatobiliary disease in dogs. Hepatocellular carcinoma (HCC) accounts for 50–70% of all hepatic tumors in dogs, also being the sixth most common cancer globally in humans (1–4). Major etiopathologies of HCC in humans are viral infections and metabolic diseases (5). HCC frequently manifests in dogs aged over 10 years, and there are anecdotal reports of over-presentation in male dogs. However, gender predisposition, specific risk factors, and the precise mechanisms of hepatocarcinogenesis are not well-characterized in dogs (6, 7). Retrospective studies revealed that Scottish Terriers with vacuolar hepatopathy, certain breeds of dogs, such as Miniature Schnauzers, Shih Tzus, Welsh Corgis, and Beagles, have high risks of developing HCC, and concurrent hyperadrenocorticism (HAC) is reportedly associated with the development of HCC (8–11). In Scottish Terriers, increased corticosteroid isoforms of alkaline phosphatase and hepatocellular vacuolation on histology are very common (12–14). HCC was detected in 34% of Scottish Terriers with vacuolar hepatopathy (8). A recent study suggested that a single nucleotide polymorphism in HSD17B2 causes an increase in adrenal sex steroids (progesterone and androstenedione) in Scottish Terriers, which results in increased alkaline phosphatase in those dogs (15, 16). Leela-Arporn et al. investigated the concurrent diseases of 44 massive HCC cases and found that 10 dogs with HCC had hypercortisolemia with HAC suggestive clinical signs (9). Thus, it is not surprising that excess adrenal steroids affect the liver and play a role in HCC development.

In humans, metabolic syndrome is a potential risk factor for non-alcoholic fatty liver disease (NAFLD) that may progress to HCC. However, the mechanism of hepatic lipid accumulation and developing HCC remains ambiguous. The clinicopathologic abnormalities of HAC, such as steroid hepatopathy caused by chronic over-production of adrenocortical hormone in dogs, somewhat resemble NAFLD-induced HCC (17, 18). An association between HAC and HCC in dogs may contribute to understand HCC development from NAFLD, a liver phenotype of the metabolic syndrome in humans. Cortisol is the main and potent glucocorticoid in dogs with HAC. Meanwhile, other adrenal steroids, such as 17α-OH progesterone, corticosterone, or 11-deoxycortisol, have been considered to cause HAC (19–21). Therefore, it may be helpful to measure multiple adrenal steroids, so-called steroid profile, to understand the link between HAC and HCC in dogs.

In veterinary medicine, steroid hormones have been traditionally measured using immunoassay because of their convenience and high sensitivity (22–24). However, the major disadvantage of immunoassay is that it is less specific due to the cross-reactivity among structurally similar steroids (25). Liquid chromatography coupled with tandem mass spectrometry (LC/MS/MS) has become more popular in clinical laboratories since LC/MS/MS can measure multiple steroid hormones at a single runtime with high specificity and sensitivity (26, 27). Although various LC/MS/MS methods have been continuously published for analyzing multiple steroid hormones in biofluids, some are particularly sensitive for a few steroids; others are feasible for detecting dozens but have poor sensitivity (28). The chemical derivative reagent is an additional sample pretreatment method that reacts to the functional groups of target analytes (amine, hydroxyl, or carbonyl groups) to enhance better ionization (29). In our previous LC/MS/MS method, it was feasible to measure the serum levels of nine steroid hormones; however, nine steroids may not be enough for the steroid profile (19). Thus, we combined the conventional non-derivatization and keto-derivatization methods for measuring multiple steroid hormones and investigated the relationship between HAC and HCC in dogs using serum steroid profiling.

## Materials and methods

### Study population

From September 2019 to December 2021, a retrospective study was conducted at the Hokkaido University Veterinary Teaching Hospital (HUVTH). A total of 46 dogs, 31 massive HCC and 15 HAC cases, were included in this study. Among the 31 massive HCC cases, 10 dogs had concurrent HAC, while 21 were HAC-free. Of the 15 dogs with HAC, abdominal ultrasonography revealed no hepatic lesion. The other endocrine disorders were 2 dogs with hypothyroidism and 1 dog with hyperparathyroidism. Myxomatous mitral valve disease (MMVD) in 5 dogs, biliary sludge in 3 dogs, chronic kidney disease (CKD) in 2 dogs, bladder stones in 2 dogs, atopic dermatitis in 2 dogs, and one each had pyoderma, mast cell tumor (MCT), hypercalcemia, and breast cancer were presented as other medical complications. Informed consent was obtained from all owners of the dogs involved in this study.

Liver tumors were collected when HCC-suspected dogs were undergoing surgery at HUVTH. Formalin-fixed liver tissues were sent to a private laboratory and HCC was histologically confirmed by a board-certificated pathologist. The pathological diagnosis of HCC was defined according to the guidelines of the World Small Animal Veterinary Association (WSAVA) Liver Standardization Group (30). Diagnostic criteria of HAC were the presence of one or more HAC-suggestive clinical signs, a positive result of the adrenocorticotropic hormone (ACTH) stimulation test, and clinical response to the trilostane treatment following 3 months (31). Six HCC dogs without HAC received corticosteroid treatment for more than 2 weeks. Those dogs were excluded from the further analysis, and serum profiling was performed on 15 dogs with HAC, 15 dogs with HCC, and 10 dogs with both diseases.

Blood of 2 ml volume was collected at their first visit to our hospital and serum was obtained by centrifuging at 3,000 rpm for 10 min. Serum was stored at −80°C until analysis of steroid profile. The breed, age, body weight, gender distribution, and neutered status of all dogs are described in Table 1.

**Table 1:** Demographic information of dogs in hepatocellular carcinoma (HCC), hyperadrenocorticism (HAC), and HCC with HAC.

	**HCC**	**HAC**	**HCC with HAC**
	***N =* 15**	***N =* 15**	***N =* 10**
Breeds (*n*)	Miniature Dachshund (2)	Miniature Dachshund (2)	Miniature Dachshund (1)
	Shiba (1)	Shiba (2)	Shiba (1)
	Miniature Schnauzer (1)	Miniature Schnauzer (1)	Miniature Schnauzer (1)
	Chihuahua (1)	Chihuahua (4)	Chihuahua (1)
	Beagle (1)	West Highland White Terrier (2)	Shih Tzu (1)
	Shih Tzu (1)	Miniature Pinscher (1)	Boston Terrier (1)
	Siberia Huskey (1)	American Cocker Spaniel (1)	Beagle (1)
	Japanese Spitx (1)	Yorkshire Terrier (1)	Mix breed (3)
	Samyoed (1)	Mix breed (1)	
	Bichon Frise (1)		
	Mix breed (4)		
Gender (*n*)	Male (1)	Male (1)	Male (0)
	Female (2)	Female (1)	Female (2)
	Neutered male (6)	Neutered male (6)	Neutered male (4)
	Spay female (6)	Spay female (7)	Spay female (4)
Median age (range) (years)	10.7 (7.2–14.8)	12.5 (5.5–14.0)	10.8 (7.7–14.3)
Median body weight (range) (kg)	7.0 (2.2–27.0)	8.7 (2.7–17.7)	9.8 (3.9–21.6)

### Chemicals and reagents

**Figure 1:**
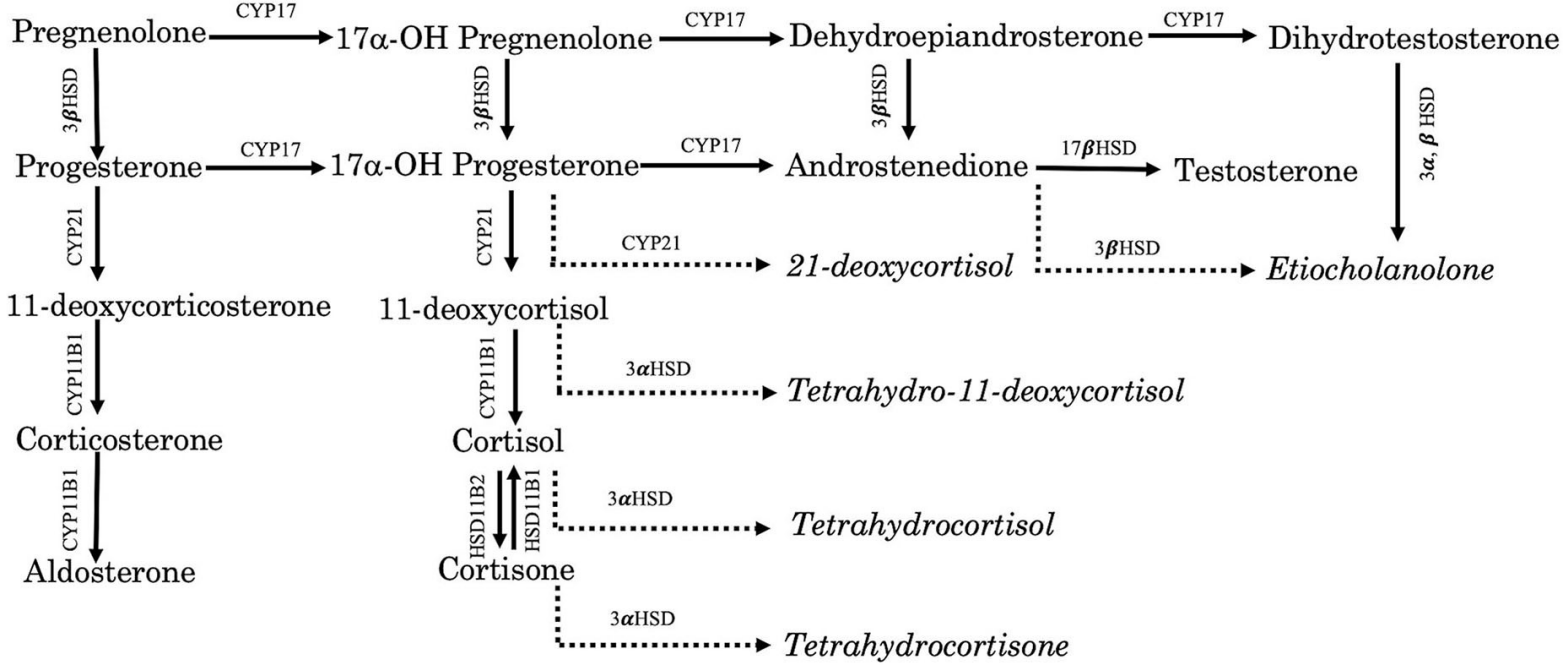
Schematic pathway of steroids and metabolites. CYP17, 17α-hydroxylase, CYP21, 21-hydroxylase, CYP 11 B1, 11β hydroxylase, 3α HSD, 3α-hydroxysteroid dehydrogenase, and 3β HSD, 3β-hydroxysteroid dehydrogenase. Italic: Metabolites.

A total of 19 steroids (Figure 1) in the serum were quantified using LC/MS/MS. The standard (STD) and stable isotope-labeled internal standard (IS) of 19 steroids were purchased from Toronto Research Chemicals (Toronto, Canada), Cerilliant (Round Rock, TX, USA), or Sigma-Aldrich (St. Louis, MO, USA). Double-distilled water (DDW) and liquid chromatography grade-analytical reagents (methanol and acetonitrile) were bought from Kanto Chemical Co., Ltd. (Tokyo, Japan). Formic acid (abt. 99%) was obtained from FUJIFILM Wako Pure Chemical Corporation (Tokyo, Japan). The phospholipid removal MonoSpin^®^ column was purchased from GL Sciences Co., Ltd. (Tokyo, Japan). Amplifex^TM^ Keto Reagent Kits were acquired from AB SCIEX Pte. Ltd. (Framingham, MA, USA). Working solutions for STD and IS mixtures were dissolved in methanol at 10 ng/ml and 100 ng/ml concentrations, respectively.

### Sample preparation

In total, 19 steroids (14 adrenal steroids and 5 metabolites) in the baseline serum of 40 dogs were quantitatively measured using two sample preparation methods. The preparation procedure is described below.

#### Non-derivatization method

First, 25 μl of serum was thoroughly mixed with 10 μl IS mixture working solution and 90 μl of 1% formic acid containing acetonitrile. The concentration of each steroid in the IS mixture was 10 ng/ml. The mixture was centrifuged at 10,000 × g for 10 min at 25°C. Afterward, 100 μl of the supernatant was applied to the MonoSpin column and centrifuged at 3,000 × g for 2 min to remove serum phospholipids. The eluate was evaporated at 60°C using a centrifugal vaporizer CVE-2000D (EYELA, Bohemia, NY, USA). Then, 100 μl of 50% methanol-double distilled water (DDW) containing 0.1% formic acid was added to reconstitute the dried tube.

#### Keto-derivatization method

First, 25 μl of serum was mixed with 10 μl of the IS mixture working solution and 90 μl of 1% formic acid in acetonitrile. The mixture was vortexed and centrifuged at 10,000 × g for 10 min. Afterward, 100 μl of the organic layer was loaded into the MonoSpin column and the phospholipids were removed by centrifuging at 3,000 × g for 2 min. The eluate was dried at 60°C using the speed vacuum evaporation. A keto-derivatization reagent was prepared according to the instructions of the manufacturer. The dried eluate was reconstituted with 50 μl of the keto-derivatization reagent and incubated for 60 min on a vortex mixer. The derivatization reaction was stopped by combining with 50 μl DDW. The final 100 μl solution was transferred to the LC/MS/MS system.

### LC/MS/MS analytical conditions

Liquid chromatography coupled with tandem mass spectrometry was performed using an Agilent 6495B Triple Quadrupole LC/MS (Agilent Technologies, Santa Clara, CA, USA) coupled with a high-performance liquid chromatography system (1260 Infinity II, Agilent Technologies). The 50 μl solution was injected into the LC/MS/MS system. Chromatographic separation was carried out on the Shim-pack Biphenyl Column (ϕ2.6 μm, 100 × 3 mm, Shimadzu, Kyoto, Japan) for the non-derivatization method and Poroshell 120/EC-C18 Column (ϕ2.7 μm, 100 × 3 mm, Agilent Technologies (L-293) Santa Clara, USA) for the keto-derivatization method, respectively. The mass spectrometry was run in the positive electrospray ionization (ESI) multiple reaction monitoring (MRM) mode. The data acquisition and processing were operated using the Agilent Mass Hunter Workstation software (Agilent Technologies).

### Validation of the method

Serum samples required for method validation were collected from 6 healthy, intact female Beagle dogs owned by the animal facility of the Graduate School of Veterinary Medicine, Hokkaido University. The method performance was determined by calculating the instrumental detection limit (IDL) and method detection limit (MDL) of the steroid hormones.

### Calibration curves

The calibration curve was created using 9 concentrations of the STD mixture (0.001, 0.005, 0.01, 0.05, 0.1, 0.5, 1, 5, and 10 ng/ml) dissolved with 1 ng/ml of the IS mixed in methanol. Peak areas of steroid hormones were manually identified with the corresponding IS peak area in each sample. The analyte peak area was less than the peak area of the lowest calibration point (0.001 ng/ml) and the concentration less than the MDL value was set as the zero concentration. The concentration of steroid hormone was calculated using the following equation.

Cs = Css × (As/Ass-b)/a

Cs: amount (ng) of target steroids

Css: amount (ng) of IS

As: Peak area of target steroids

Ass: Peak area of IS

a: slope of the calibration curve

b: intercept of the calibration curve

### Statistical analysis

Statistical analyses were performed using commercial software (JMP Pro^®^16, version 16.0.0, SAS Institute Inc, Cary, NC, USA). A comparison of age and body weight was carried out using the Kruskal-Wallis test. Gender distribution and detection rate of steroid hormones among the groups were analyzed using the chi-square test. Principle component analysis (PCA) was performed to see the steroid pattern difference between the disease groups. The Steel-Dwass test was used for a non-parametric multiple comparison of median steroid concentration among HCC, HAC, and HCC with HAC dogs. *p*-value (< 0.05) was considered statistically significant for all comparisons.

## Results

Breeds were not over-presented in 15 dogs with HCC, 15 dogs with HAC, and 10 HCC with HAC dogs (Table 1). Age, body weight, and gender distribution were not significantly different between the groups (*p* > 0.05).

A gradient program and column settings are described in Tables 2A,B for the non-derivatization and keto-derivatization methods, respectively. The mass-to-charge transition of 19 steroids and ISs in positive MRM mode are presented in Tables 3A,B for the non-derivatization and keto-derivatization methods, respectively. The assay performance, i.e., IDL and MDL values of each steroid, is described in Table 4.

**Table 2A:** Gradient program and column temperature in the non-derivatization method.

**Pump system**	**Mobile phase A**	**0.1% formic acid in DDW**	
	**Mobile phase B**	**0.1% formic acid in methanol**	
Temperature		50°C	
**Gradient condition pump system**	**Mobile phase A (%)**	**Mobile phase B (%)**	**Flow rate (ml/min)**
0 min	60.00	40.00	0.4
0.50 min	60.00	40.00	0.4
0.51 min	60.00	40.00	0.8
3.00 min	60.00	40.00	0.8
19.00 min	15.00	85.00	0.8
19.50 min	0.00	100.00	0.8
21.00 min	0.00	100.00	0.8
21.01 min	60.00	40.00	0.4
**Ion source**	**Sheath gas temp (** **°** **C)**	**Sheath gas flow rate (L/min)**	**Drying gas temperature (** **°** **C)**	**Drying gas flow rate (L/min)**	**Nebulizer (psi)**	**Capillary (V)**
Electrospray Ionization	350.00	12.00	290	19	40	3,500

**Table 2B:** Gradient program and column temperature in the keto-derivatization method.

**Pump system**	**Mobile phase A**	**0.1% formic acid in DDW**	
	**Mobile phase B**	**0.1% formic acid in methanol**	
Temperature		50°C	
**Gradient condition pump system**	**Mobile phase A (%)**	**Mobile phase B (%)**	**Flow rate (mL/min)**
0 min	65.00	35.00	0.4
1.50 min	65.00	35.00	0.4
1.51 min	65.00	35.00	0.8
17.00 min	40.00	60.00	0.8
17.01 min	0.00	100.00	0.8
18.50 min	0.00	100.00	0.8
18.51 min	65.00	35.00	0.4
**Ion source**	**Sheath gas temp (** **°** **C)**	**Sheath gas flow rate (L/min)**	**Drying gas temperature (** **°** **C)**	**Drying gas flow rate (L/min)**	**Nebulizer (psi)**	**Capillary (V)**
Electrospray Ionization	350.00	12.00	290	17	220	3,500

**Table 3A:** Multiple reaction monitoring conditions of steroids and their internal standards in the non-derivatization method.

**Native STD/Stable isotope labeled IS**	**Precursor ion (*m/z*)**	**Quantifier** **(*m/z*)**	**Qualifier** **(*m/z*)**	**Collision energy (V) quantifier**	**Collision energy (V) qualifier**	**Polarity**
						
Progesterone	315.2	97.0	108.9	24	28	+
Progesterone-d9	325.0	100.1	113.2	20	32	+
17α -OH progesterone	331.2	109.1	97.2	28	44	+
17α -OH progesterone-13C3	334.0	100.2	81.2	32	68	+
Cortisol	363.2	121.1	91.0	32	68	+
Cortisol-d4	367.4	121.0	97.1	24	52	+
Cortisone	361.2	163.0	91.1	25	70	+
Cortisone-d8	369.2	168.2	93.4	20	72	+
Corticosterone	347.2	121.2	91.0	28	68	+
Corticosterone-d4	351.4	121.0	97.1	44	40	+
11-deoxycorticosterone	331.5	109.2	97.0	32	20	+
11-deoxycorticosterone-13C3	334.1	100.0	112.1	24	28	+
11-deoxycortisol	347.2	97.1	109.0	32	28	+
11-deoxycortisol-d5	352.0	100.3	113.1	28	28	+
21-deoxycortisol	347.5	91.1	311.6	72	16	+
21-deoxycortisol-d8	355.3	319.3	125.2	16	24	+
Aldosterone	361.2	343.3	91.1	20	80	+
Aldosterone-d4	365.3	347.2	97.0	20	36	+
Androstenedione	287.2	97.1	109.1	20	24	+
Androstenedione−13C3	290.0	100.0	112.0	28	28	+
Testosterone	289.2	97.1	109.0	20	28	+
Testosterone-13C3	292.0	100.1	111.9	28	20	+

**Table 3B:** Multiple reaction monitoring conditions of steroids and their internal standards in the keto-derivatization method.

**Native STD/Stable isotope labeled IS**	**Precursor ion (*m/z*)**	**Quantifier** **(*m/z*)**	**Qualifier** **(*m/z*)**	**Collision energy (V) quantifier**	**Collision energy (V) qualifier**	**Polarity**
						
Pregnenolone	431.4	372.2	126.1	24	44	+
Pregnenolone-d4	435.5	376.3	130.1	28	48	+
17α -OH pregnenolone	447.3	370.2	388.2	28	20	+
17α -OH pregnenolone-13C3d2	451.4	374.2	392.4	28	24	+
Etiocholanolone	405.5	346.2	91.1	28	72	+
Etiocholanolone-d5	410.3	351.2	105.0	16	72	+
Dehydroepiandrosterone	403.4	344.2	105.1	28	68	+
Dehydroepiandrosterone-d5	408.0	349.2	162.2	28	48	+
Dihydrotestosterone	405.4	346.2	91.1	32	80	+
Dihydrotestosterone-d3	408.5	349.2	81.1	24	60	+
Tetrahydrocortisol	481.0	118.1	116.0	56	44	+
Tetrahydrocortisol-d5	486.0	118.2	60.1	48	68	+
Tetrahydrocortisone	479.0	118.0	59.1	40	64	+
Tetrahydrocortisone-d5	484.0	118.0	116.1	52	40	+
Tetrahydro-11-deoxycortisol	465.0	118.1	59.1	48	80	+
Tetrahydro-11-deoxycortisol-d5	470.0	118.1	115.9	44	44	+

**Table 4:** Assay performance of the non-derivatization and keto-derivatization methods.

**Detection method**	**Steroid hormones**	**IDL (pg)**	**MDL (ng/ mL)**
Non-derivatization method	Cortisol	0.02	0.13
	Cortisone	0.02	0.18
	11-deoxycortisol	0.01	0.06
	11-deoxycorticosterone	0.02	0.02
	Corticosterone	0.02	0.04
	Progesterone	0.01	0.01
	17α-OH progesterone	0.01	0.09
	Androstenedione	0.03	0.09
	Testosterone	0.01	0.19
	21-deoxycortisol	0.01	0.03
	Aldosterone	0.06	0.06
Keto-derivatization method	Pregnenolone	0.02	0.03
	17α-OH pregnenolone	0.02	0.02
	Tetrahydrocortisol	0.02	0.03
	Tetrahydrocortisone	0.04	0.02
	Tetrahydro-11-deoxycortisol	0.01	0.02
	Dehydroepiandrosterone	0.03	0.04
	Dihydrotestosterone	0.04	0.01
	Etiocholanolone	0.02	0.06

The detection rate of steroid hormones in the non-derivatization and keto derivatization methods in dogs with HCC, HAC, and both diseases are described in Table 5. 21-Deoxycortisol and aldosterone were detected in one-fourth of the total samples. Androstenedione and dehydroepiandrosterone were hardly detected around 10%. Etiocholanolone was detected only in one HAC dog while testosterone and dihydrotestosterone were detected in each dog of the HCC with the HAC group. The detection rate of 19 steroids was not significantly different between groups (*p* > 0.05). Steroid hormones with a detection rate of higher than 50% were selected for further statistical analysis.

**Table 5:** Detection rate (%) of steroid hormones in the non-derivatization and keto-derivatization methods.

**Detection method**	**Steroid hormones**	**Overall dogs (*N =* 40)**	**HCC** **(*N =* 15)**	**HAC** **(*N =* 15)**	**Both diseases (*N =* 10)**	**Chi Square (*p* < 0.05)**
Non-derivatization method	Progesterone (%)	95 (38/40)	93 (14/15)	100 (15/15)	90 (9/10)	NS
	17α-OH progesterone (%)	68 (27/40)	67 (10/15)	67 (10/15)	70 (7/10)	NS
	11-deoxycorticosterone (%)	86 (34/40)	80 (12/15)	93 (14/15)	80 (8/10)	NS
	11-deoxyortisol (%)	100 (40/40)	100 (15/15)	100 (15/15)	100 (10/10)	NS
	Corticosterone (%)	100 (40/40)	100 (15/15)	100 (15/15)	100 (10/10)	NS
	Cortisol (%)	100 (40/40)	100 (15/15)	100 (15/15)	100 (10/10)	NS
	Cortisone (%)	100 (40/40)	100 (15/15)	100 (15/15)	100 (10/10)	NS
	Aldosterone (%)	25 (10/40)	27 (4/15)	27 (4/15)	20 (2/10)	NS
	21-deoxycortisol (%)	35 (14/40)	20 (3/15)	47 (7/15)	40 (4/10)	NS
	Androstenedione (%)	13 (5/40)	13 (2/15)	20 (3/15)	0	–
	Testosterone (%)	5 (2/40)	7 (1/15)	7 (1/15)	0	–
Keto-derivatization method	Pregnenolone (%)	100 (40/40)	100 (15/15)	100 (15/15)	100 (10/10)	NS
	17α-OH pregnenolone (%)	78 (31/40)	80 (12/15)	73 (11/15)	80 (8/10)	NS
	Tetrahydrocortisol (%)	93 (37/40)	87 (13/15)	93 (14/15)	100 (10/10)	NS
	Tetrahydrocortisone (%)	100 (40/40)	100 (15/15)	100 (15/15)	100 (10/10)	NS
	Tetrahydro-11-deoxycortisol (%)	48 (19/40)	33 (5/15)	40 (6/15)	80 (8/10)	NS
	Dehydroepiandrosterone (%)	10 (4/40)	13 (2/15)	13 (2/15)	0	–
	Dihydrotestosterone (%)	5 (2/40)	7 (1/15)	7 (1/15)	0	–
	Etiocholanolone (%)	3 (1/40)	0	7 (1/15)	0	–

**Figure 2:**
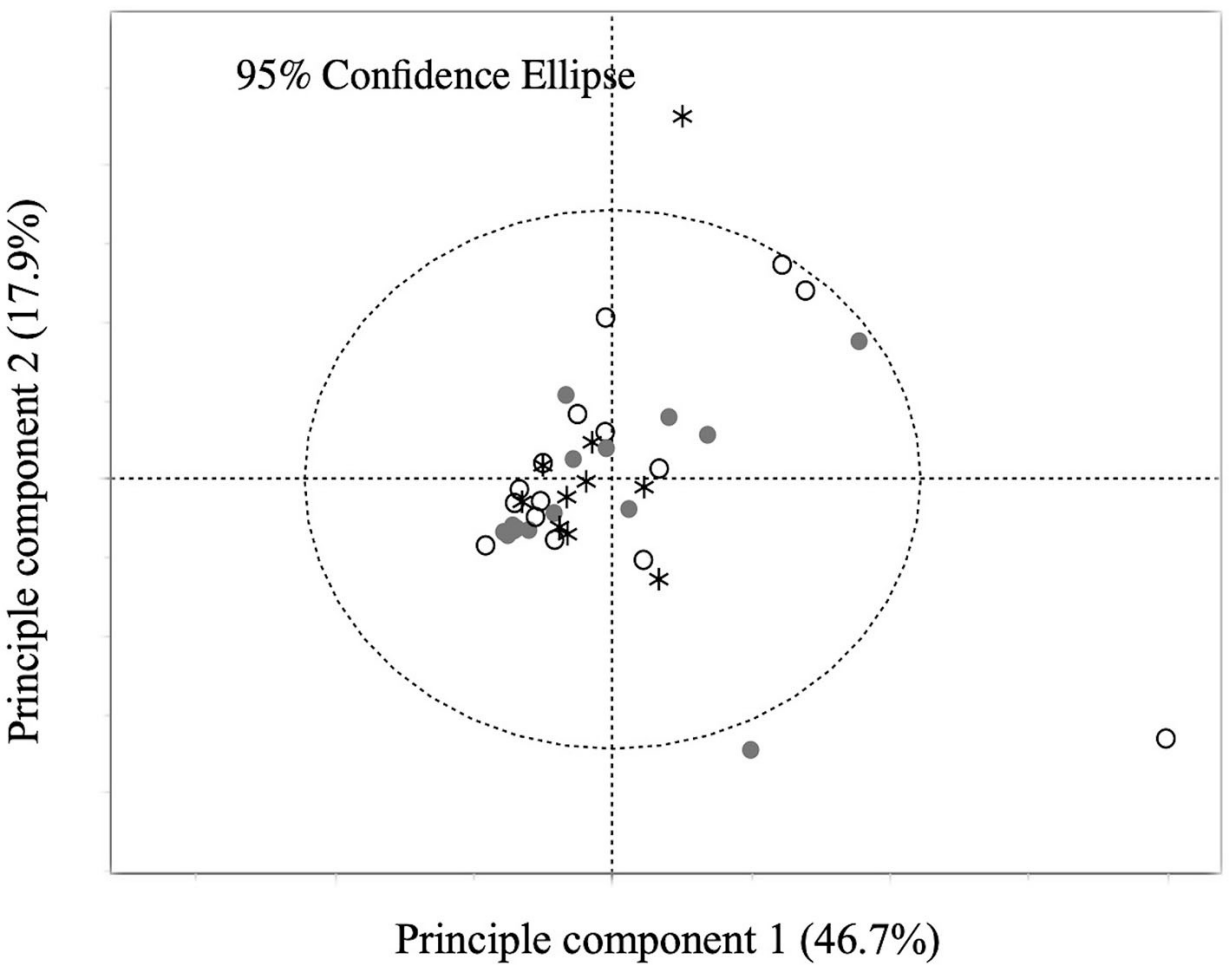
Principal component analysis (PCA) of the concentration of 11 steroids. Points labeled in white circles are dogs with HCC, in gray circles are dogs with HAC, and in asterisk are dogs with both diseases.

**Figure 3:**
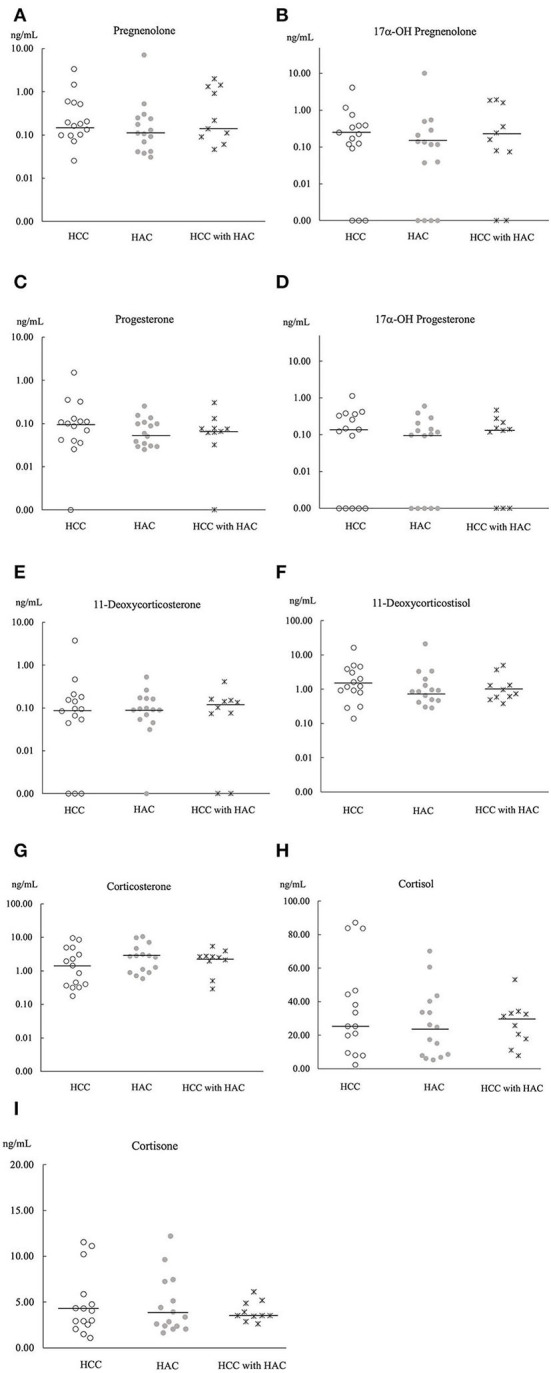
Comparison of 9 steroids concentration **(A–I)** in baseline serum of HCC (*n* = 15), HAC (*n* = 15), and HCC with HAC dogs (*n* = 10). Bars; the median concentration of steroids in each group.

**Figure 4:**
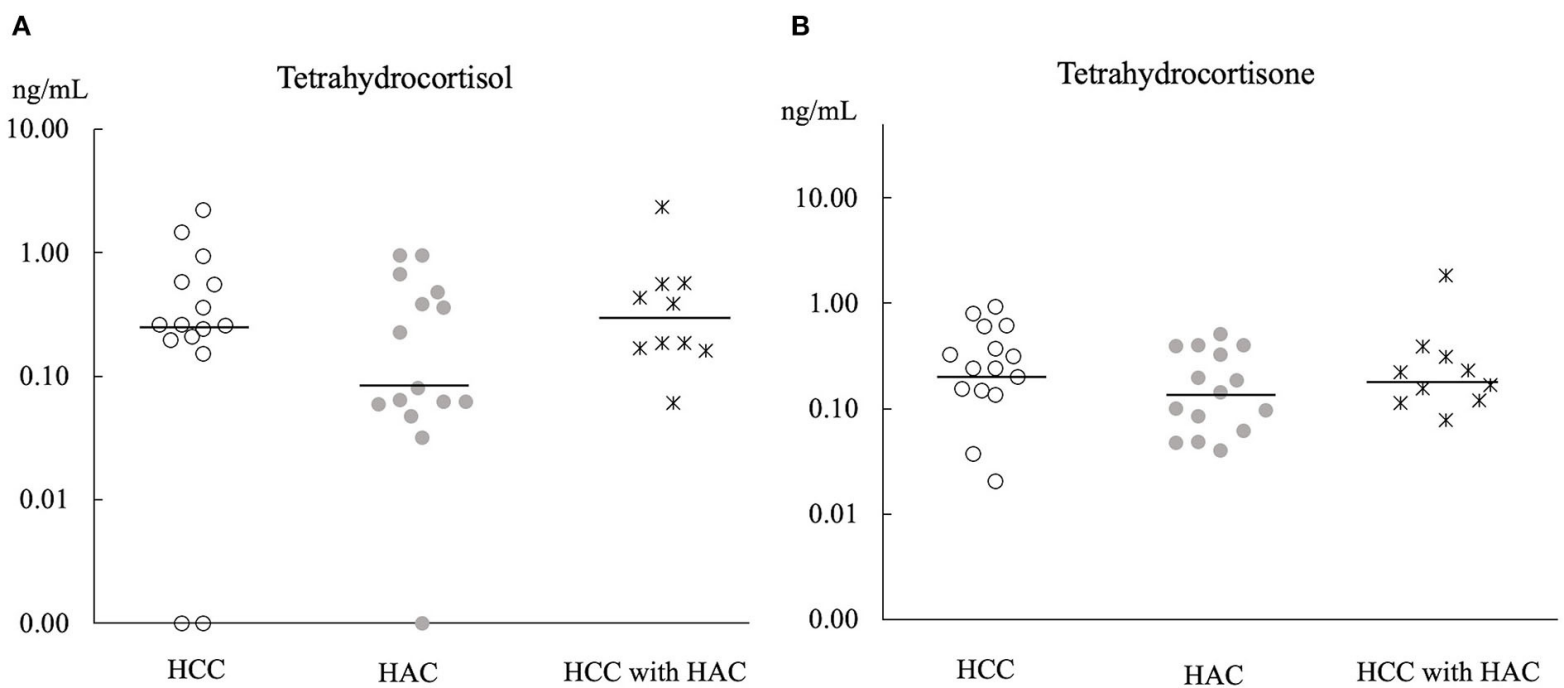
Comparison of 2 metabolites concentration **(A,B)** in baseline serum of HCC (*n* = 15), HAC (*n* = 15), and HCC with HAC dogs (*n* = 10). Bars; the median concentration of steroids in each group.

Principle component analysis explains no steroid pattern difference among the groups (Figure 2). Figures 3, 4 compare the median concentrations of 9 steroids and 2 metabolites, respectively. None of the 11 steroid concentrations significantly differed between dogs with HCC, HAC, and both diseases (*p* > 0.05).

## Discussion

In this study, steroid profiling of the baseline serum was performed to clarify the relationship between HCC and HAC. The co-occurrence of massive HCC and HAC was comparable, 32% (10/31) in this study and 23% (10/44) in a previous study (9). However, the baseline serum steroid profiling at the time of diagnosis was failed to find the association between HCC and HAC.

Liver cirrhosis and fibrosis caused by viral infections are the major causes of HCC development in humans but are rarely reported in dogs. Moreover, NAFLD occurs in 20% of people with Cushing's disease and precedes HCC development because steroid hormones cause multi-hit theories in Cushing patients with NAFLD (32). Excess glucocorticoids stimulate gluconeogenesis in the liver and consequently cause hepatic steatosis, steatohepatitis, and fibrosis in humans (33). Although hepatopathy with glycogen accumulation was found in dogs with HAC, steroid-induced hepatic inflammation, progressive findings of liver cirrhosis, or fibrosis, such as human HCC, seemed not to be preceded in dogs (34). Other confounding factors, such as changes in expression of steroidogenic factors, genetic mutation or epigenetic changes, and inflammatory cytokines, are thought to influence the occurrence of HAC in dogs with HCC. To our knowledge, there is only one online dissertation report on TP 53 gene mutation in canine HCC (35).

Chronic exposure to exogenous or endogenous glucocorticoid hormones is considered to cause vacuolar hepatopathy, which is a potential risk of developing HCC (33). Furthermore, adrenocortical hormone, concentrations are expected to be high in dogs with HAC because of hyperfunctions of the adrenal glands (31). However, a comparison of 11 steroid hormones (Figures 3, 4) did not show a significant difference between dogs with HCC, HAC, and both diseases. Our findings indicate that steroid profile may not be involved in HCC development, although there was a noticeable concurrence of HAC and HCC. Sepesy et al. support our findings that 45% of vacuolar hepatopathy occurred without endogenous or exogenous glucocorticoid exposure in dogs (14). In Scottish terriers, vacuolar hepatopathy dogs show HAC suggestive signs with changes in non-cortisol hormones. However, some vacuolar hepatopathy dogs developed HCC with or without HAC suggestive signs and non-cortisol changes (8). Even in Scottish terriers, the exact mechanism of steroidogenesis disorders in the progression of vacuolar hepatopathy to HCC has been unclear.

Aldosterone and 21-deoxycortisol were detected in one-third of the samples with low concentration. In the canine mineralocorticoid pathway, zonal expression of aldosterone synthase is limited to a single gene (CYP11B1), with no expression of a zone-specific enzyme (CYP11B2) when compared with humans (16). Androgen concentration in many dogs was measured under the MDL value. The current study included only 7 intact dogs; the remaining 33 were neutered or spayed. Even though androgens can activate the oncoprotein transcription in human HCC and neuter status might be a risk factor for some malignancies in dogs, current findings suggest that androgens may not be involved in the development of dog HCC (36–38).

Additionally, even though our results do not support the idea that steroidogenesis changes may impact on the development of hepatocarcinogenesis, the coincidence of HAC in HCC is a concern. HCC may also play a role in the steroid imbalance as an alternative to our hypothesis. Hepatocarcinogenesis may disturb steroidogenesis or steroid metabolism in the liver. Steroids and metabolites in liver tissues and 24-h urine samples may be useful to confirm metabolic changes inside the liver in future analysis. This study only evaluated serum steroids at a single time point and was unable to determine when HCC or HAC first started in dogs. Therefore, follow-up case studies were warranted to prove the alternative theory.

The reliable measurement of steroid hormones is a powerful technique to investigate their hormonal status in endocrine-related diseases. By combining the elution effect and a biphenyl column, the technique provided sufficient column separation and symmetric peaks for isobaric steroids, such as 21-deoxycortisol, 11-deoxycortisol, and corticosterone (39). Following the non-derivatization preparation steps, derivatization with the keto-reagent is simple, fast, and sensitive when compared with other different derivatization strategies (40–42). The MDL values were acceptable, and the present LC/MS/MS method could be used to measure multi-steroid hormones (Table 4).

To conclude our study, we developed the LC/MS/MS method for measuring multiple steroid hormones in a small amount of serum. The concentration of 19 serum steroids was not different in dogs with HCC, HAC, and both diseases. We concluded that HAC is not associated with the development of HCC, and steroidogenesis may not contribute to hepatocarcinogenesis. Additional measurement of steroids inside the liver and metabolites from the liver should be used to conduct more in-depth research on steroid imbalance in hepatocarcinogenesis.

## Data availability statement

The original contributions presented in the study are included in the article/Supplementary material, further inquiries can be directed to the corresponding author.

## Ethics statement

The animal study was reviewed and approved by Laboratory Animal Experimentation Committee of the Graduate School of Veterinary Medicine, Hokkaido University (Approval No. 18-0142). Written informed consent was obtained from the owners for the participation of their animals in this study.

## Author contributions

TO: conceptualization, formal analysis, investigation, data curation, visualization, writing-original draft, and project administration. NS: conceptualization, writing-original draft, review, and editing. YI: methodology, validation, and funding acquisition. TI: methodology and validation. NN: investigation. NY, KS, and KM: resources. KN: supervision. MT: conceptualization, writing-review and editing, supervision, project administration, and funding acquisition. All authors have read and agreed to the published version of the manuscript.

## Funding

This study was supported by the Grant-in-Aid for Scientific Research from the Japanese Society for the Promotion of Science (YI; JP18H04132, JP22K18425, JP22K05980 and MT; JP20H03139) and the MEXT project for promoting public utilization of advanced research infrastructure (program for supporting the introduction of the new sharing system, JPMXS0420100619). TO was supported by the World Leading Innovative and Smart Education (WISE) program (1801) from the Ministry of Education, Culture, Sports, Science, and Technology, Japan.

## Conflict of interest

The authors declare that the research was conducted in the absence of any commercial or financial relationships that could be construed as a potential conflict of interest.

## Publisher's note

All claims expressed in this article are solely those of the authors and do not necessarily represent those of their affiliated organizations, or those of the publisher, the editors and the reviewers. Any product that may be evaluated in this article, or claim that may be made by its manufacturer, is not guaranteed or endorsed by the publisher.
